# Novel pseudo-random number generator based on quantum random walks

**DOI:** 10.1038/srep20362

**Published:** 2016-02-04

**Authors:** Yu-Guang Yang, Qian-Qian Zhao

**Affiliations:** 1 College of Computer Science and Technology, Beijing University of Technology, Beijing, 100124, China; 2 State Key Laboratory of Information Security (Institute of Information Engineering, Chinese Academy of Sciences, Beijing, 100093), China; 3 Beijing Key Laboratory of Trusted Computing, Beijing, 100124, China; 4 National Engineering Laboratory for Critical Technologies of Information Security Classified Protection, Beijing, 100124, China

## Abstract

In this paper, we investigate the potential application of quantum computation for constructing pseudo-random number generators (PRNGs) and further construct a novel PRNG based on quantum random walks (QRWs), a famous quantum computation model. The PRNG merely relies on the equations used in the QRWs, and thus the generation algorithm is simple and the computation speed is fast. The proposed PRNG is subjected to statistical tests such as NIST and successfully passed the test. Compared with the representative PRNG based on quantum chaotic maps (QCM), the present QRWs-based PRNG has some advantages such as better statistical complexity and recurrence. For example, the normalized Shannon entropy and the statistical complexity of the QRWs-based PRNG are 0.999699456771172 and 1.799961178212329e-04 respectively given the number of 8 bits-words, say, 16Mbits. By contrast, the corresponding values of the QCM-based PRNG are 0.999448131481064 and 3.701210794388818e-04 respectively. Thus the statistical complexity and the normalized entropy of the QRWs-based PRNG are closer to 0 and 1 respectively than those of the QCM-based PRNG when the number of words of the analyzed sequence increases. It provides a new clue to construct PRNGs and also extends the applications of quantum computation.

Random numbers have an extensive application in various contexts including statistical mechanics, gaming industry, cryptography and communication etc. Two basic types of random number generators exist: true random number generators (TRNGs) and pseudo-random number generators (PRNGs). Generally, the generation of TRNGs depends on certain physical sources such as thermal noise^1^, atmospheric noise^2^, radioactive decay^3^, etc. Although TRNGs are considered to attain a higher security, the implementation of TRNGs generally requires additional devices which make TRNGs inconvenient^4^.

By contrast, PRNGs can generate “pseudo-random” numbers deterministically by inputting an initial seed to given algorithms. The main advantages of PRNGs are the rapidity and the repeatability of the pseudo-random sequences and requiring less memory for algorithm storage. In general, PRNGs are based on certain mathematical difficulty assumptions, such as: non-linear congruences^5^, linear feedback shift registers (LFSR)^6^, discrete logarithm problem^7^, quadratic residuosity problem^8^, cellular automata^9^,^10^, etc. Unfortunately, such PRNGs are usually slower, due to heavy computational instructions.

Another interesting way to design PRNGs is connected to chaos theory^11^. Chaotic systems are characterized by their high sensitivity to initial conditions and some properties like ergodicity, pseudo-random behavior and high complexity^11^ which make chaotic systems very attractive for implementing PRNGs. Several PRNGs have been proposed^12^,^13^,^14^,^15^. However, some security loopholes often occur in such chaos-based PRNGs due to the lack of rigorous security analyses^16^.

As one of the most important contributions in nonlinear science, classical chaos theory has been studied widely and applied in various contexts such as mathematics, physics, chemistry, computer science, biology and so on. Quantum information theory has achieved a rapid development due to the fascinate quantum effects such as quantum superposition and entanglement. A natural question that arises is how to characterize chaos in the quantum regime, i.e., how to manifest chaos itself at the quantum level. This has led to the development of quantum chaos theory. Signatures of chaos in quantum systems have been explored in the contexts of level statistics of chaotic Hamiltonians^17^, the dynamics of open quantum systems undergoing measurement or decoherence^18^,^19^ and hypersensitivity of a system to perturbations^20^,^21^.

Another natural question that arises is how to use the chaotic characteristics of such quantum chaotic systems. In fact, the uses of quantum chaos in constructing PRNGs and other applications have been explored^22^,^23^,^24^,^25^. For many years dissipative quantum maps were widely used as informative models of quantum chaos, such as the quantum kicked top, the quantum baker’s map, the quantum lazy baker’s map, and the quantum sawtooth and cat maps^26^,^27^. It is natural to ask whether there exist other quantum chaotic systems with more excellent chaotic behaviours.

Quantum computation is a rapidly growing field and lots of breakthroughs have been achieved during the past decades^28^. As a universal quantum computation model, QRWs are the quantum counterparts of classical random walks and have been developed as a useful tool for solving various problems^29^,^30^,^31^,^32^, such as element distinctness, finding the triangle, and routing, etc. Furthermore, the widespread application of classical random walks in many fields like physics, biology, computer science, finance, etc., infers the possibility that its quantum analog, namely, QRWs, could be used as a tool for many future applications.

Inspired by the above reasons, we are motivated to search for novel quantum chaotic systems. In this paper, we investigate QRWs and propose a novel QRWs-based PRNG. It is found that the QRWs-based PRNG can generate more excellent pseudo-randomness than the QCM-based PRNG^22^ by numerical simulations and performance comparisons in terms of quantifiers based on information theory, recurrence plots, non-periodity, and various randomness tests, etc.

## Results

### The chaotic behavior of quantum random walks

QRWs have two models: discrete QRWs and continuous QRWs^28^. The one-dimensional (1D) discrete QRWs on the line includes two quantum systems: a walker whose motion is restricted to the line and a coin. The state of the walker-coin system is denoted by a vector in the Hilbert space 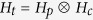, where the subscripts *p* and *c* stand for walker and coin, respectively. The motion of the walk is conditioned by the coin state via a conditional shift operator


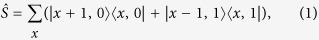


where the summation symbol denotes the sum over all possible positions. The evolution of the total quantum system can be implemented by repeating the sequence of the coin flipping operator and the conditional shift operator in equation (1) step by step (so-called discrete time), expressed by


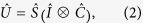


where 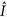 is the identity operator of the walker and 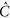 is the flipping operator applied to the coin state, generally expressed by 
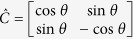
, 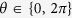. Hence the final state 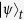 after *t* steps is expressed by


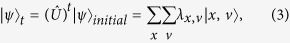


and the probability of locating the walker at position *x* after *t* steps is


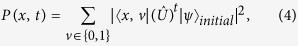


where 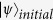 is the initial state of the total quantum system.

For *n*-dimensional discrete QRWs on the line, the final state 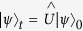 after *t* steps is expressed by


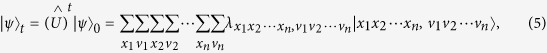


and the probability of locating the *n* walkers at position 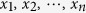 after *t* steps is


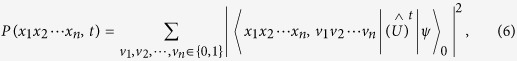


where 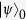 is the initial state of the total *n*-walker, *n*-coin quantum system. It can be seen that the resulting probability distribution in equation (6) is the sum of squares of the norms of amplitudes so that there exists a non-linearity map between the initial state 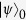 and the resulting probability distribution. And the high sensitivity to initial conditions underlies the proposed PRNG.

### Pseudo random number generator based on quantum random walks

In this section, we discuss how to construct the QRWs-based PRNG by running the one-dimensional discrete QRWs on a circle. In the one-dimensional discrete QRWs on a circle with *N* nodes, the position state 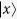 should be altered to 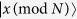. The steps of generating pseudo-random numbers are as follows:

1. Choose the initial parameters (*N*, (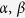), *r*, 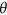) of the one-dimensional discrete QRWs on a circle with *N* nodes and run it to generate a probability distribution 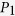. Here *r* is the step number of the QRWs whose value belongs to the positive integer domain. *N* is the node number of the circle whose value also belongs to the positive integer domain. 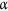 and 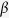 are the amplitude parameters of the coin states which are complex numbers and satisfy the constraint: 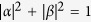. 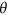 is the parameter of the coin operator 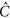, where 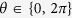. Here,

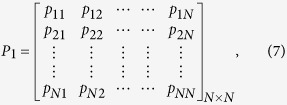


where 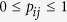. We transform the probability distribution 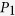 into the sequence 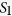: [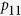, 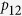, 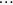

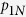
, 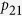, 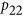, 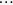

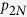
, 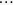, 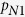, 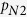, 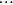

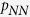
].

1. Repeat step (1) and group all generated probability distribution sequences 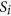
(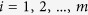) into a random number sequence 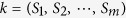.

Note that quantum walks on the line have a property that when a walker takes *r* steps, where *r* is an odd number, the probability of standing on a point that labeled even is zero. When *r* is even, the probabilities on odd points are zero too. But on an odd circle with *N* nodes, when 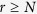, the probabilities on all nodes are nonzero essentially. Therefore, we cannot judge the number of steps, even its parity.

### Security analyses of the QRWs-based PRNG

Experiments are performed on a laptop with Intel(R) Core(TM)2 Duo CPU T5870 2.00 GHz RAM running on Windows 7 professional equipped with the MATLAB R2012a environment. Here we chose the initial key parameters (*N* = 6, (*α* = 0, *β* = 1), *r* = 10, *θ* = *π*/3).

In order to measure the randomness of the QRWs-based PRNG, some quantifiers were proposed. The quantifiers are mainly classified into two classes: (i) quantifiers based on information theory^33^,^34^,^35^, (ii) quantifiers based on recurrence plots^36^,^37^.

#### Statistical complexity measure

**Figure 1:**
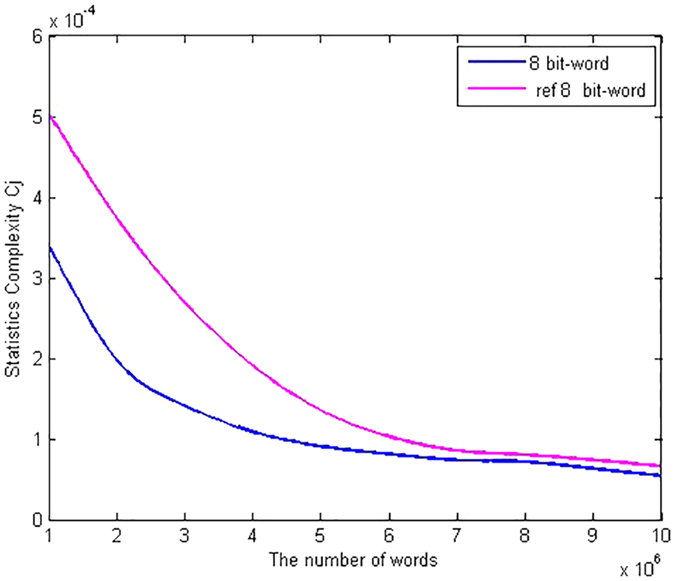
Comparisons in terms of Normalized Shannon entropy 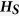
. The red curve denotes the Normalized Shannon entropy in the QCM-based scheme^22^ as functions of the number of 8 bits-words, while the blue curve represents the Normalized Shannon entropy of our proposal. (see text in the section entitled Results).

**Figure 2:**
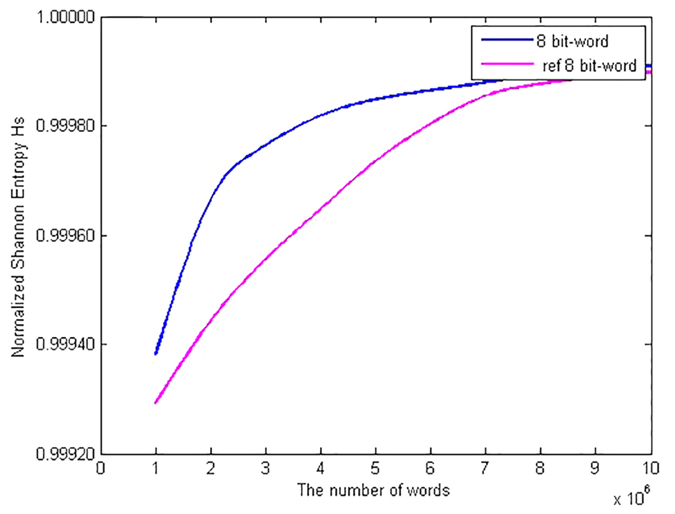
Comparisons in terms of intensive statistical complexity measure 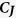
. The red curve denotes the intensive statistical complexity in the QCM-based scheme^22^ as functions of the number of 8 bits-words, while the blue curve represents the intensive statistical complexity of our proposal. (see text in the section entitled Results).

Complexity is a measure of off-equilibrium ‘order’. Statistical complexity measures (SCM) were proposed as quantifiers of the degree of physical structure in a signal^33^,^38^,^39^. They are null for total random processes. In this section, based on the method of ref. 40, we analyzed the statistical complexity of the QRWs-based PRNG. The intensive SCM (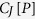) can be considered as a quantity that characterizes the probability distribution *P* associated with the time series generated by the dynamical system^40^. It quantifies not only randomness but also the presence of correlational structures^39^,^40^ and can be used to study the intricate structures hidden in the dynamics. The measure of statistical complexity 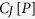 is defined as^40^:


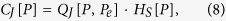


where the normalized entropic measure 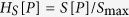 is associated with the probability distribution *P*, with 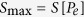
(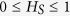) for the equilibrium distribution 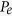 and *S* is the Shannon entropy. The disequilibrium 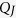 is defined in terms of the Jensen-Shannon divergence^40^ by


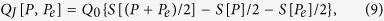


with 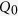 being the normalization constant (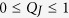). Thus, the disequilibrium 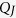 is an intensive quantity. Following the methodology proposed by Bandt and Pompe^41^, the comparisons between our proposal and the QCM-based scheme^22^ in terms of the normalized entropy 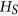 and the intensive statistical complexity 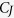 as functions of the number of 8 bits-words are shown in Figs 1 and 2 respectively. As can be seen from the figures, when the number of words of the analyzed sequence increases, the statistical complexity and the normalized entropy tend to 0 and 1 respectively. It is shown that given the same words, our scheme has better statistical complexity and normalized Shannon entropy than the PRNG scheme based on QCM^22^. For example, the normalized Shannon entropy and the statistical complexity of the QRWs-based PRNG are 0.999699456771172 and 1.799961178212329e-04 respectively given the number of 8 bits-words, say, 16Mbits. By contrast, the corresponding values of the QCM-based PRNG are 0.999448131481064 and 3.701210794388818e-04 respectively. Thus the statistical complexity and the normalized entropy of the QRWs-based PRNG are closer to 0 and 1 respectively than those of the QCM-based PRNG when the number of words of the analyzed sequence increases. It can be concluded that, the randomness of the proposed QRWs-based PRNG is successfully verified by the statistical complexity and the normalized Shannon entropy.

#### Recurrence plots

Recurrence is a fundamental property of dynamical systems, which can be exploited to characterize the system’s behaviour in phase space. In 1987, Eckmann *et al*. introduced a powerful tool for visualization and analysis of recurrences called recurrence plot (*RP*)^36^. *RP* is a two-dimensional representation in which both axes are time ones. The recurrence of a state appearing at two given times 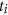, 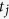 is pictured in the two-dimensional graph by means of either black or white dots, where a black dot denotes a recurrence.

To visualize the recurrences of states of a dynamical system, the *RP* of a trajectory 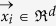 can be formally expressed by the matrix


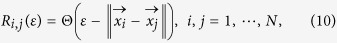


where *N* is the number of measured points 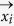, 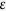 is a threshold distance, 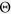
(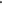) is the Heaviside function (i.e. 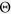
(*x*) = 0, if *x* <0, and 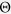
(*x*)=1 otherwise) and 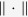 is a norm.

Because the visual impact produced by the *RP* is insufficient to demonstrate the quality of the QRWs-based PRNG because of the ‘small-scale’ structures^37^, several measures of complexity which quantify the small scale structures in *RP*s, have been proposed^42^,^43^,^44^ and are known as recurrence quantification analysis (*RQA*). In this paper, these measures based on the diagonal and vertical line structures are considered.

#### Measures based on diagonal lines

The measures are related to the histogram 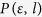 of the diagonal line lengths *l*, given by


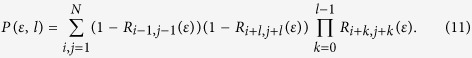


The ratio of recurrence points that form diagonal structures (of at least length 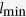) to all recurrence points is defined by


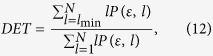


as a measure for determinism (or predictability) of the system. The threshold 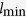 excludes the diagonal lines which are formed by the tangential motion of the phase space trajectory.

A diagonal line of length *l* means that a segment of the trajectory is rather close during *l* time steps to another segment of the trajectory at a different time; thus these lines are related to the divergence of the trajectory segments. The average diagonal line length


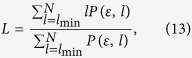


is the average time that two segments of the trajectory are close to each other, and can be interpreted as the mean prediction time.

#### Measures based on vertical lines

The total number of the vertical lines of the length *v* in the *RP* is then given by the histogram


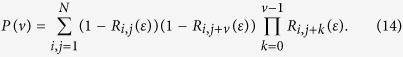


Analogous to the definition of the determinism in equation (12), the ratio between the recurrence points forming the vertical structures and the entire set of recurrence points can be computed,


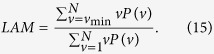


The computation of *LAM* is realized for those *v* that exceed a minimal length 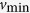 in order to decrease the influence of the tangential motion. *LAM* will decrease if the *RP* consists of more single recurrence points than vertical structures.

The average length of vertical structures is given by


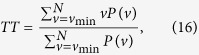


and is called trapping time. *TT* estimates the mean time that the system will abide at a specific state or how long the state will be trapped.

**Figure 3:**
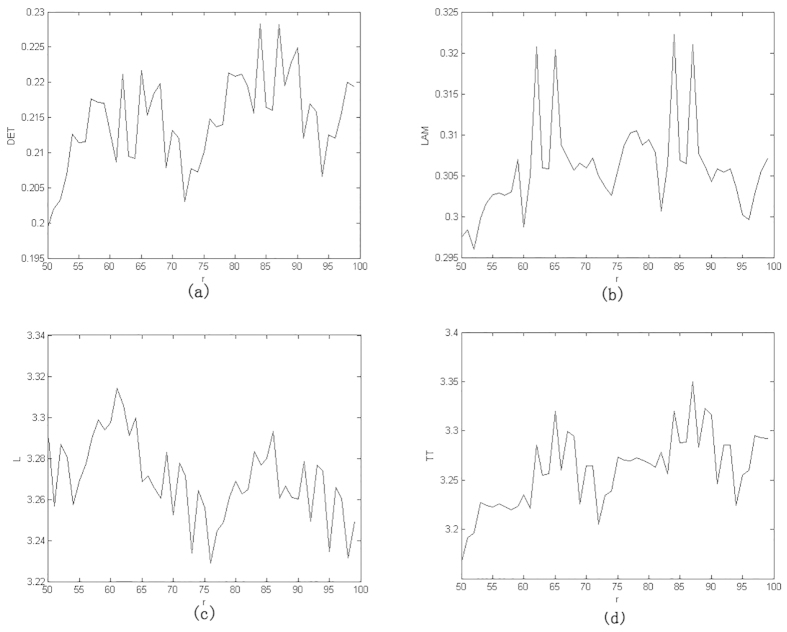
Selected *RQA* measures for the QRWs-based PRNG. (**a**) *DET*, (**b**) *LAM*, (**c**) *L*, (**d**) *TT*. (see text in the section entitled Results).

**Figure 4:**
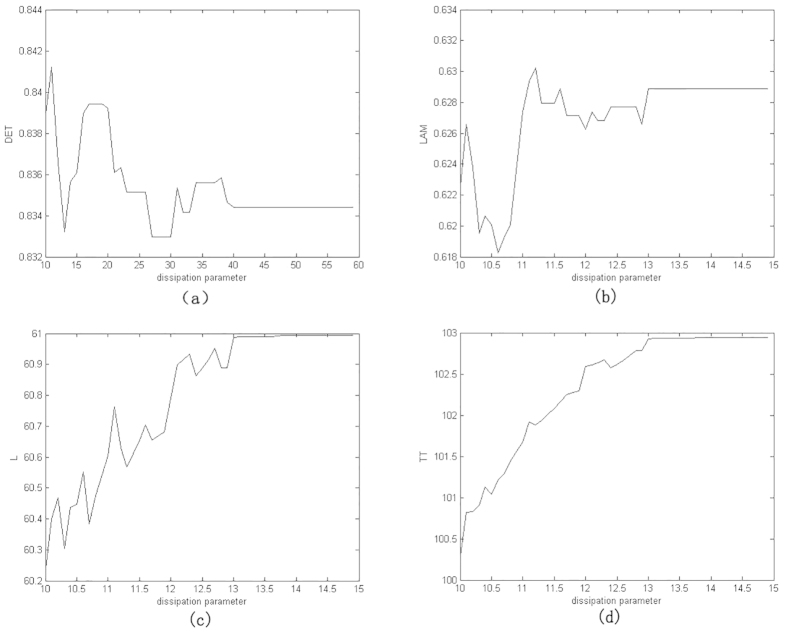
Selected *RQA* measures for the QCM-based scheme 22. (**a**) *DET*, (**b**) *LAM*, (**c**) *L*, (**d**) *TT*. (see text in the section entitled Results).

Figure 3 gives some selected *RQA* measures for different values of the parameter *r* and demonstrates the good statistical properties of the QRWs-based PRNG. Figure 4 gives the corresponding selected RQA measures for different values of the dissipation parameter 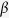 based on QCM^25^ with the initial parameters (*x*,*y*,*z*,*r*) = (0.62352345,0.0152345,0.0352345,3.99). Generally, processes with uncorrelated or weakly correlated and stochastic or chaotic behaviours cause none or very short diagonals, whereas deterministic processes cause longer diagonals (verticals) and less single, isolated recurrence points. Therefore, the measure *DET* or *LAM* should be small given an appropriate threshold 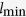 (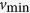), with a typical value of 0.3 or so as shown in Fig. 3. The measure *L* or *TT* is the average time that two segments of the trajectory are close to each other, and can be interpreted as the mean prediction time, with a typical value of 3 or so given the parameters at hand. By contrast, the corresponding values of the QCM-based PRNG, i.e., *DET, LAM, L* and *TT* average 0.8, 0.6, 60 and 100, respectively. It can be concluded that, the randomness of the proposed QRWs-based PRNG is successfully verified.

#### Degree of non-periodicity

In this section, we use the scale index to study the non-periodicity in the QRWs-based PRNG, which is introduced by Benìtez *et al*.^45^. The scale index technique is based on the continuous wavelet transform (CWT) and the wavelet multi-resolution analysis^46^. To study non-periodicity of the QRWs-based PRNG^47^, we assumed that the key sequence *f* is compactly supported and defined over a finite time interval *I* = [*a, b*]. In order to avoid boundary problems, the wavelet function is compactly supported and the interval *I* is big enough.

The CWT of *f* at time *u* and scale *s* is defined as follows^46^:


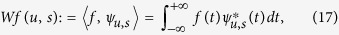


and it provides the frequency component (or details) of *f* corresponding to the scale *s* and time location *t*.

The scalogram of *f* is defined as follows:


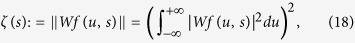


where 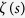 is the energy of the CWT of *f* at scale *s*. The scalogram is a useful tool for studying a signal, since it allows the detection of its most representative scales or frequencies^45^,^47^. Also, the inner scalogram of *f* at a scale *s* can be defined by:


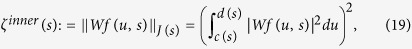


where 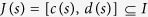 is the maximal subinterval in *I* for which the support of 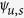 is included in *I* for all 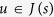. As the length of 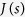 depends on the scale *s*, the values of the inner scalogram at different scales cannot be compared. Therefore, the inner scalogram should be normalized as follows^45^:


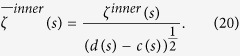


Supplementary Figure S1 online shows that the normalized inner scalogram can be a valuable tool for detecting the non-periodicity of the signal, where a signal with details at every scale is non-periodic. Here the selection of the scale interval 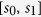 is very important in the scalogram analysis. Since the non-periodic character of a signal is given by its behavior at large scales, there is no need for 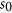 to be very small. In general, we can choose 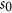 such that 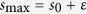 where 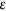 is positive and close to zero. On the other hand, 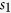 should be large enough for detecting periodicities. Here, we considered the integer scales between 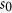 = 1 and 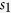 = 20.

The scale index of *f* in the scale interval 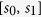 can be defined by:


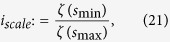


where 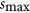 is the smallest scale such that 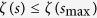 for all 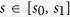, and 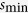 is the smallest scale such that 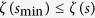 for all 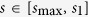. Note that for compactly supported signals only the normalized inner scalogram will be considered^45^. From its definition, the scale index 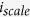 meets 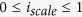 and it can be interpreted as a measure of the degree of non-periodicity of the signal: the scale index will be zero or close to zero for periodic sequences and close to one for highly non-periodic sequences^45^.

**Figure 5:**
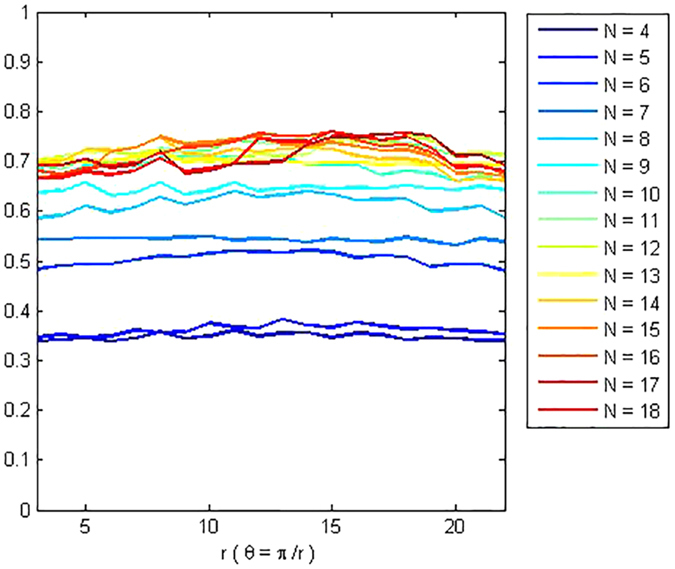
**The scale index of the QRWs-based PRNG for different values of the parameter** ***θ.***

Since the scale index gives a measure of the degree of non-periodicity of the signal, this can be used to specify which values of the QRWs parameters are best for the generation of pseudo-random number sequences. In Fig. 5, the scale index analysis of the QRWs-based key sequence is presented. It can be concluded that, the best value of the scale index is 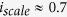 for all 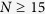 and 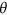, which is just the upper bound of that of the scheme in ref. 22. Thus, the sequence in this state is highly non-periodic and it can be used for any PRNG purpose.

#### Key space analysis

A desirable image encryption scheme should have a sufficiently large key space to resist brute-force attacks. The encryption key of our algorithm can be represented by (*n*, (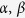), *r*, 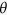). Although there is an infinite key space theoretically, because of finite precision of digital computers, the key space actually turns out to be finite. Considering that the calculation precision is 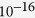, the size of key space for initial conditions and control parameters would be roughly 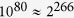, which is large enough for any encryption purposes and is also large enough to resist all kinds of brute-force attacks.

#### Random tests for the pseudo-random sequences

To verify the randomness property of our QRWs-based PRNG sequence, we used NIST SP800-22 to test the randomness of the sequences generated by QRWs (see Table 1). Each test produces a *P-value* in [0, 1]. If the *P-value* is higher than the preset threshold 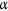, it means that the sequences pass the test. In our tests, we set *α* = 0.01 and generate a large of number to meet the requirements of the software NIST for the magnitude 1000000. *α* = 0.01 implies that the pseudo-random sequence can be inferred to be random with 99% probability if it passes the test. From Table 1, the results of different number sequences generated by QRWs are all “success”. Hence, we can judge that our QRWs-based generator passes the NIST SP800-22 tests.

#### Speed performance analysis

Speed is an important factor for evaluating the performance of a PRNG. For the proposed PRNG algorithm, we measured the time cost in the running environment: Windows 7, Matlab R2012a, Intel(R) Core(TM) i3-2370M CPU 2.00GHz 2GB RAM and the mean time cost of generating random sequences is 0.001361s or so. Therefore, our algorithm is fast enough for practical application.

## Discussion

As a kind of TRNGs, quantum random number generators (QRNGs) can significantly improve the security of cryptographic protocols by means of quantum effects. QRNGs have typically been based on specialized physical hardware, such as single-photon sources and detectors^48^ or homodyne detection^49^, photon-number resolving detectors^50^, parametric oscillators^51^, or Raman scattering^52^. However, the cost, size, and power requirements of current QRNGs have prevented them from becoming widespread. Fortunately, Sanguinetti *et al*.^53^ proposed a novel method for quantum random number generation by means of cameras which can be integrated in many common devices such as cell photons, tablets, and laptops. They exploited the quantum effect called “quantum noise” or “shot noise” to realize a QRNG by using a detector capable of resolving this distribution. In experiment, Sanguinetti *et al*. exploited the image sensors in cameras and smartphones to resolve quantum noise. In contrast to Sanguinetti *et al*.’s QRNG, our QRWs-based PRNG exploits the properties of QRWs, i.e., the high sensitivity of the walker’s position probability distribution to initial conditions. Without any physical device, the PRNG merely relies on the equations used in the QRWs, and thus the random number generation algorithm is simple and the computation speed is fast.

As a quantum analog of simulated annealing, quantum annealing (QA) has also attracted lots of attention^54^,^55^. It can be exploited for solving optimization problems by using quantum tunneling. In QA, the optimization problem is encoded in a Hamiltonian 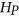. The algorithm starts by introducing strong quantum fluctuations by adding a disordering Hamiltonian 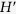 that does not commute with 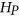. An example case is


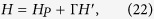


where 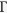 changes from a large value to zero during the evolution. The disorder is slowly removed by removing 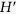
(reducing 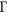). Generally, it is rather difficult to solve the Schrödinger equation in equation (22) and thus the random-process-based methods are used, such as quantum Monte Carlo method. If the process is slow enough, the system will settle in a local minimum close to the exact solution. Theoretically the slower the evolution, the better the solution will be achieved at the cost of consuming longer computation time. To reduce the computation time, on the one hand, QRWs can be introduced into the moving strategies for performing Metropolis Monte Carlo sampling so as to decrease the times of finding global optimum efficiently using the properties of quantum parallel computation. On the other hand, the QRWs-based PRNG can also be used for quantum annealers as an efficient random number generator needed during iteration.

## Conclusion

In a summary, we have proposed a new QRWs-based PRNG. Numerical simulations demonstrate that the proposed PRNG exhibits excellent pseudo-randomness in terms of quantifiers based on information theory, recurrence plots, non-periodity, and NIST tests. It can be concluded that the new QRWs-based PRNG can generate a high percentage of usable pseudo-random numbers for various applications and it also extends the application scope of quantum computation.

Future work will also concentrate on how to design QRWs with better cryptographic properties. To obtain more complicated non-linear dynamic behaviors of QRWs, it is necessary to research the construction of multi-walker, multi-coin QRWs on a circle, tree, graph or other graph structures.

## Methods

To generate the probability distribution 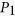, we assume the initial state of the total quantum system of the one-dimensional discrete QRWs on a circle with *N* nodes


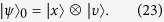


Here


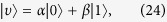


where 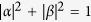.

The difference between a line and a circle is that the circle has *N* nodes and is cyclical. The difference of walks on the line and on circles is that the conditional shift operator on circles becomes 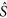, i.e.,


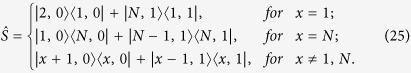


Here we let the coin operator 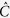



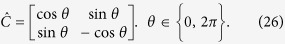


Choose the initial parameters (*N*, (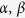), *r*, 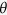) of the one-dimensional discrete QRWs on a circle with *N* nodes and run it to generate a probability distribution 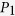, given by


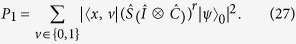


## Additional Information

**How to cite this article**: Yang, Y.-G. and Zhao, Q.-Q. Novel pseudo-random number generator based on quantum random walks. *Sci. Rep.* **6**, 20362; doi: 10.1038/srep20362 (2016).

## Supplementary Material

Supplementary Information

## Figures and Tables

**Table 1:** Randomness test by NIST SP800-22 for the QRWs-based PRNG.

Test name	P-value	Result
Approximate entropy test (block = 10)	0.565844	SUCCESS
Block frequency test (block = 128)	0.932368	SUCCESS
Cumulative sums (forward) test	0.438435	SUCCESS
Cumulative sums (reverse) test	0.051895	SUCCESS
Spectral DFT test	0.180314	SUCCESS
Frequency test	0.222465	SUCCESS
Linear complexity (block = 500)	0.826735	SUCCESS
Longest runs of ones test	0.388167	SUCCESS
Non-Overlapping templates test		
(m = 9, template = 000111101)	0.962460	SUCCESS
Overlapping template of all ones test (m = 9)	0.577368	SUCCESS
Random excursions test (x = −1)	0.506488	SUCCESS
Random excursions variant test (x = +1)	0.527057	SUCCESS
Rank test	0.436267	SUCCESS
Runs test	0.436267	SUCCESS
Serial test1 (block = 16)	0.719705	SUCCESS
Serial test2 (block = 16)	0.580439	SUCCESS
Universal statistical test (block = 7)	0.596355	SUCCESS
