# Supplementary material for: Novel pseudo-random number generator based on quantum random walks
Source: Sci Rep. 2016 Feb 4;6:20362. doi: 10.1038/srep20362 (PMC4740897; doi:10.1038/srep20362)
Supplement: Supplementary Information [file srep20362-s1.pdf]

Supplemental materials

Novel pseudo-random number generator based on quantum random walks

Yu-Guang Yang, Qian-Qian Zhao

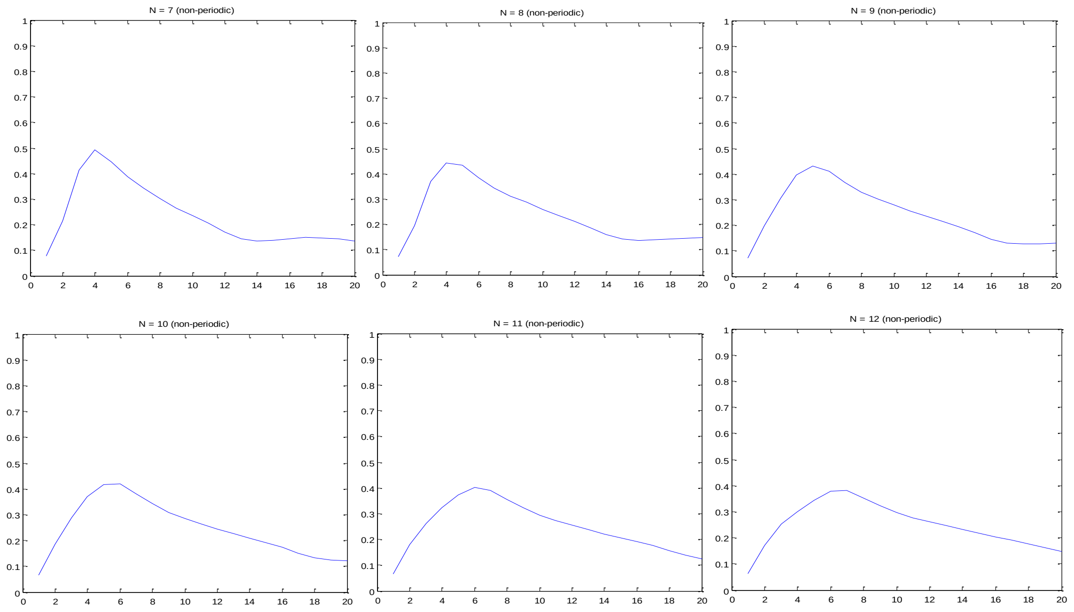

Supplementary Figure S1| Normalized inner scalograms for the QRW-based key sequence *f* with the scale parameter *s* running from s_0 =1 to s_1 =20, with Δs =0.05, and using the Daubechies eight-wavelet function.
